# Post-Stroke Rehabilitation: Challenges and New Perspectives

**DOI:** 10.3390/jcm12020550

**Published:** 2023-01-10

**Authors:** Giovanni Morone, Floriana Pichiorri

**Affiliations:** 1 Department of Life, Health and Environmental Sciences, University of L’Aquila, 67100 L’Aquila, Italy; 2 San Raffaele Institute of Sulmona, 67039 Sulmona, Italy; 3 Neuroelectrical Imaging and Brain–Computer Interface Laboratory, Fondazione Santa Lucia IRCCS, 00179 Rome, Italy

A stroke is determined by insufficient blood supply to the brain due to vessel occlusion (ischemic stroke) or rupture (hemorrhagic stroke), resulting in immediate neurological impairment to differing degrees. Due to its etiology, it is prevalent among the elderly population even though its impact on young adults is possibly higher given the longer life expectancy of survivors. Stroke is the leading cause of disability worldwide and its incidence will increase along with the aging population. On one hand, improvements in acute stroke care (fibrinolytic therapy or endovascular treatment) aim to reduce the burden of residual neurological damage. On the other hand, efficient medical management of early phase complications (e.g., infections) will hopefully result in an increased number of stroke survivors. 

Thus, neurorehabilitation remains crucial in determining the personal and societal burden of stroke consequences in the medium to long term. These range from sensorimotor impairment affecting the person’s ability to stand, walk or properly use the upper limbs to attend to the activities of daily life, cognitive impairment including speech disturbances, impaired swallowing and more. These factors, together with the management of comorbidities, stroke-related epilepsy, and sleep disturbances, all impact on the patient’s quality of life and social participation after the event. 

In this multifaceted scenario, clinicians and researchers working in post-stroke rehabilitation in the last decade have produced a considerable amount of evidence for successfully assessing post-stroke consequences and have suggested treatment approaches with different degrees of technological complexity. This has resulted in a further increase in the number of characters composing the multidisciplinary rehabilitation team, now including bio-engineers and physicists besides the physicians nurses, therapists, and psychologists from several specialties.

The vast amount of work is reflected in European stroke rehabilitation guidelines [1,2,3] which now mention technology-based therapies for cognitive and motor rehabilitation alongside traditional indications on early mobilization, constraint-induced movement therapy, task-oriented repetitive training and aerobic exercises. The management of swallowing impairment, which leads to malnutrition and poor stroke outcomes [4] is also underlined in most rehabilitation guidelines and has now reached possibly the highest level of published evidence in the field [5]. 

Despite these advancements there is still little consensus on which approach is the most effective for each category of patient, among the plethora of novel solutions including those based on robotics, non-invasive brain stimulations, [6] brain–computer interfaces [7], and more. In other words, while many of these approaches have proven some level of efficacy, even in well-designed randomized controlled trials (RCTs), most patients are offered these options according to their availability in the facilities that they refer to for rehabilitation with the certainty that they will do no harm and in the presumption that they will contribute to a better outcome.

There is a tremendous need for patient stratification in order to direct resources to patients who will benefit most from a given rehabilitation approach. To reach this goal, researchers should pursue a trade-off between large RCTs and improvements in longitudinal personalized approaches [8]. On one hand, large numbers are needed in order to overcome the intrinsic variability in the spontaneous recovery after a stroke. On the other hand, variability should be deeply investigated with the very intent of identifying markers of response to a given treatment, in order to improve the personalization of neurorehabilitation pathways. In this context, the advancements made in assessing specific deficits and in measuring specific outcomes via neuroimaging, neurophysiology and other advanced bioengineering techniques (i.e., robots and sensors) will hopefully lead to the identification of potential novel markers of good recovery. Needless to say, the achievements in the field of post-stroke rehabilitation will inevitably depend on the successful integration of different professionals, representing a unique opportunity for multidisciplinarity.

In the light of this scenario, with the aim of evaluating the efficacy of a specific therapy for the motor and cognitive recovery of patients with neurological disease, the aggregation of numerical data (as done in systematic reviews) is not always useful to deduce the dilemma. An example of this is a recent review of systematic reviews of robotics which showed that in the face of primary studies of excellent quality, most of the systematic reviews lack sufficient methodological quality with few exceptions [9].

It is fair to say that technological devices have now entered neurorehabilitation wards, at least in high-income countries [10]; however, efforts must be made to direct these interventions to the best responding categories of patients and possibly extend these benefits to mid- and low-income countries [11]. To reach this goal, extensive longitudinal assessments and defining measurable outcomes is paramount, and it must be directed to evaluate the benefits of rehabilitation in terms of actual improvements in daily life activities, i.e., the improvements must be clinically and functionally relevant to justify the investment of resources. 

A further challenge that the neurorehabilitative community will have to face in the future concerns the great need for chronic care. Indeed, in the absence of an increase in devoted economic resources, the outpatient setting will not be able to respond adequately to such needs. It will likely be necessary to rethink the patient’s home as a place of care. In this sense, telemedicine and telerehabilitation have proven effective during periods of confinement (in the recent SARS-CoV-2 pandemic) and for remote rural areas, but could eventually become a resource to be added to chronic rehabilitation facilities [12]. The potential of telerehabilitation could also be effective in reducing the uneven availability of advanced treatment options, even in high-income countries (e.g., in peripheral and rural areas). These instruments could be used to identify, via remote assessments, candidates for specific interventions and thus eventually justify the logistical efforts on behalf of the patients, caregivers and healthcare providers. Additionally, this would apply to all geographic areas facing conflicts, natural disasters and other possible causes of isolation which are unfortunately very relevant nowadays. All in all, the post-stroke neurorehabilitation field is a complex and multifaceted one, requiring different skills and knowledge from clinicians and non-clinical specialists. To face this complexity, professionals willing to work in this field must be provided with adequate learning opportunities and specific training which is currently lacking in formal education programs, e.g., nurses, therapists and even physicians. Efforts are being made in this sense on behalf of national and international scientific societies in this field, which foster multidisciplinarity and integration with neighboring fields. However, there is still a wide gap between the research context and the everyday clinical practice. This gap must be filled with the contributions from formal educational institutions, clinics and government regulations to foster translationality, evenly distributed resources and optimized efforts. 

## References

[1] Quinn T.J., Richard E., Teuschl Y., Gattringer T., Hafdi M., O’Brien J.T., Merriman N., Gillebert C., Huyglier H., Verdelho A. (2021). European Stroke Organisation and European Academy of Neurology joint guidelines on post-stroke cognitive impairment. Eur. Stroke J..

[2] Ahmed N., Audebert H., Turc G., Cordonnier C., Christensen H., Sacco S., Sandset E.C., Ntaios G., Charidimou A., Toni D. (2018). Consensus statements and recommendations from the ESO-Karolinska Stroke Update Conference, Stockholm 11–13 November. Eur. Stroke J..

[3] https://isa-aii.com/linee-guida-spread-viii-edizione/.

[4] Ciancarelli I., Morone G., Iosa M., Cerasa A., Calabrò R.S., Iolascon G., Gimigliano F., Tonin P., Tozzi Ciancarelli M.G. (2022). Influence of Oxidative Stress and Inflammation on Nutritional Status and Neural Plasticity: New Perspectives on Post-Stroke Neurorehabilitative Outcome. Nutrients.

[5] Dziewas R., Michou E., Trapl-Grundschober M., Lal A., Arsava E.M., Bath P.M., Clavé P., Glahn J., Hamdy S., Pownall S. (2021). European Stroke Organisation and European Society for Swallowing Disorders guideline for the diagnosis and treatment of post-stroke dysphagia. Eur. Stroke J..

[6] Morone G., Capone F., Iosa M., Cruciani A., Paolucci M., Martino Cinnera A., Musumeci G., Brunelli N., Costa C., Paolucci S. (2022). May Dual Transcranial Direct Current Stimulation Enhance the Efficacy of Robot-Assisted Therapy for Promoting Upper Limb Recovery in Chronic Stroke?. Neurorehabilit. Neural Repair.

[7] Pichiorri F., Mattia D. (2020). Brain-computer interfaces in neurologic rehabilitation practice. Handb. Clin. Neurol..

[8] Micera S., Caleo M., Chisari C., Hummel F.C., Pedrocchi A. (2020). Advanced Neurotechnologies for the Restoration of Motor Function. Neuron.

[9] Straudi S., Baluardo L., Arienti C., Bozzolan M., Lazzarini S.G., Agostini M., Aprile I., Paci M., Casanova E., Marino D. (2022). Effectiveness of robot-assisted arm therapy in stroke rehabilitation: An overview of systematic reviews. NeuroRehabilitation.

[10] Morone G., Paolucci S., Mattia D., Pichiorri F., Tramontano M., Iosa M. (2016). The 3Ts of the new millennium neurorehabilitation gym: Therapy, technology, translationality. Expert Rev. Med. Devices.

[11] Owolabi M.O., Platz T., Good D., Dobkin B.H., Ekechukwu E.N.D., Li L. (2020). Editorial: Translating Innovations in Stroke Rehabilitation to Improve Recovery and Quality of Life Across the Globe. Front. Neurol..

[12] Laver K.E., Adey-Wakeling Z., Crotty M., Lannin N.A., George S., Sherrington C. (2020). Telerehabilitation services for stroke. Cochrane Database Syst. Rev..

